# Comparison of Non-Tumoral Portal Vein Thrombosis Management in Cirrhotic Patients: TIPS Versus Anticoagulation Versus No Treatment

**DOI:** 10.3390/jcm10112316

**Published:** 2021-05-26

**Authors:** Chenyang Zhan, Vinay Prabhu, Stella K. Kang, Clayton Li, Yuli Zhu, Sooah Kim, Sonja Olsen, Ira M. Jacobson, Nabil N. Dagher, Brendan Carney, Ryan M. Hickey, Bedros Taslakian

**Affiliations:** 1 Division of Vascular Interventional Radiology, Department of Radiology, NYU Grossman School of Medicine, New York, NY 10016, USA; chenyang.zhan@nyulangone.org (C.Z.); Clayton.Li@nyulangone.org (C.L.); Yuli.Zhu@nyulangone.org (Y.Z.); Ryan.Hickey@nyulangone.org (R.M.H.); 2 Division of Abdominal Imaging, Department of Radiology, NYU Grossman School of Medicine, New York, NY 10016, USA; Vinay.Prabhu@nyulangone.org (V.P.); Stella.Kang@nyulangone.org (S.K.K.); Sooah.Kim@nyulangone.org (S.K.); 3 Department of Population Health, NYU Grossman School of Medicine, New York, NY 10016, USA; 4 Hepatology Section, Division of Gastroenterology, Department of Medicine, NYU Grossman School of Medicine, New York, NY 10016, USA; Sonja.Olsen@nyulangone.org (S.O.); Ira.Jacobson@nyulangone.org (I.M.J.); 5 Transplant Institute, Department of Surgery, NYU Grossman School of Medicine, New York, NY 10016, USA; Nabil.Dagher@nyulangone.org; 6 Department of Radiology, University of Iowa Hospitals and Clinics, Iowa City, IA 52242, USA; bcarney@nyit.edu

**Keywords:** non-tumoral portal vein thrombosis, transjugular intrahepatic portosystemic shunt, anticoagulation

## Abstract

Background: There is a lack of consensus in optimal management of portal vein thrombosis (PVT) in patients with cirrhosis. The purpose of this study is to compare the safety and thrombosis burden change for cirrhotic patients with non-tumoral PVT managed by transjugular intrahepatic portosystemic shunt (TIPS) only, anticoagulation only, or no treatment. Methods: This single-center retrospective study evaluated 52 patients with cirrhosis and non-tumoral PVT managed by TIPS only (14), anticoagulation only (11), or no treatment (27). The demographic, clinical, and imaging data for patients were collected. The portomesenteric thrombosis burden and liver function tests at early follow-up (6–9 months) and late follow-up (9–16 months) were compared to the baseline. Adverse events including bleeding and encephalopathy were recorded. Results: The overall portomesenteric thrombosis burden improved in eight (72%) TIPS patients, three (27%) anticoagulated patients, and two (10%) untreated patients at early follow-up (*p* = 0.001) and in seven (78%) TIPS patients, two (29%) anticoagulated patients, and three (17%) untreated patients in late follow-up (*p* = 0.007). No bleeding complications attributable to anticoagulation were observed. Conclusion: TIPS decreased portomesenteric thrombus burden compared to anticoagulation or no treatment for cirrhotic patients with PVT. Both TIPS and anticoagulation were safe therapies.

## 1. Introduction

Non-tumoral portal vein thrombosis (PVT) is an important complication of cirrhosis, with a prevalence ranging between 0.6% and 26% among patients with cirrhosis [1,2,3,4]. PVT contributes to portal hypertension [2,5] and is associated with multiple complications including intestinal ischemia, variceal bleeding, liver decompensation, and ascites [5,6,7]. Furthermore, the presence of PVT is associated with higher rates of morbidity and mortality from liver transplants in this patient population [7,8,9].

Anticoagulation therapy has frequently been utilized to treat non-tumoral PVT in cirrhosis, and high recanalization rates up to 93% have been reported [1,10,11]. While some data suggest that anticoagulation therapy is safe in cirrhotic patients with PVT with similar rates of bleeding complications as untreated patients [1,10], there are still concerns regarding its safety in cirrhotic patients who are at higher risk of hemorrhagic complications, particularly related to portal hypertension, thrombocytopenia, and coagulopathy [12]. In addition, there is a lack of consensus in the optimal management of PVT in cirrhotic patients, particularly regarding the choice of anticoagulation and therapy duration. Management with anticoagulation is largely dependent on expert opinions, clinician’s experience, and consensus during multidisciplinary meetings [13,14].

Transjugular intrahepatic portosystemic shunt (TIPS), a procedure aiming to restore main PV flow, has been demonstrated to help resolve PVT effectively without anticoagulation by relieving flow stasis [7,15]. However, TIPS has generally been reserved for pre-transplant patients [7,16]. TIPS was recommended for liver transplant candidates with progression of PVT not responsive to anticoagulation by the EASL guideline [12], but there is still no direct comparison of TIPS alone versus anticoagulation alone in the management of PVT.

The aim of our study was to retrospectively compare the safety and change in degree of portomesenteric venous thrombosis in cirrhotic patients with non-tumoral PVT when managed with anticoagulation only, TIPS only, or no treatment.

## 2. Materials and Methods

### 2.1. Study Cohort

**Figure 1:**
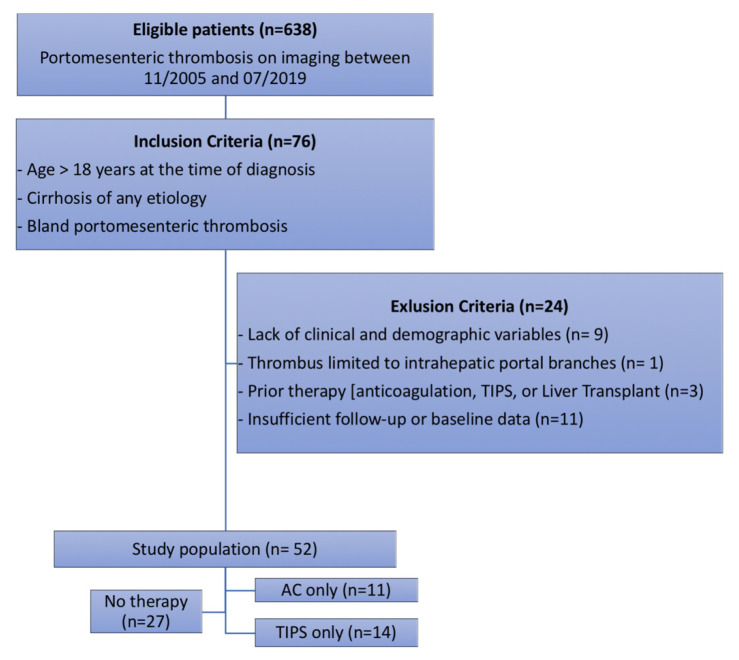
Study design flowchart.

This single-center retrospective study was Institutional Review Board (IRB) approved and Health Insurance Portability and Accountability Act (HIPAA) compliant. The electronic medical records of 638 consecutive patients presenting with imaging diagnosis of portomesenteric thrombosis between November 2005 and July 2019 at our academic tertiary referral hospital were reviewed. A total of 76 adult patients (age > 18) with cirrhosis of any etiology and imaging diagnosis of non-tumoral portomesenteric thrombosis were included (Figure 1). Portomesenteric thrombosis was defined as partial or complete absence of intraluminal flow in the main portal vein (PV), superior mesenteric vein (SMV), and/or splenic vein (SV) on contrast-enhanced magnetic resonance imaging (MRI) or computed tomography (CT). Non-tumoral portomesenteric thrombosis was defined as the lack of enhancement or having similar signal intensities or extensions to an adjacent parenchymal tumor. After excluding 24 patients based on exclusion criteria, a total of 52 patients (age 60.4 ± 10.8 years; male 77%) were included in the study (Figure 1). All patients were followed up with until death, liver transplantation (LT), or the end of the study (December 2019). In our institution, complex hepatobiliary cases are discussed in weekly multidisciplinary conferences, and management plans are made based on consensus. Based on the management of portomesenteric thrombosis, patients were categorized into the following management groups: (a) no therapy (27 patients; 52%), (b) anticoagulation only (11 patients; 21%), and (c) TIPS only (14 patients; 27%). Day 0 was defined as the date of diagnosis in the untreated group and the date of treatment in the TIPS and anticoagulation groups to reduce the influence from immortal time bias [17].

### 2.2. Clinical and Treatment Data

Demographics and clinical data recorded from electronic medical records included age, gender, BMI, alcohol use, ascites, and the presence of esophageal and gastric varices. The laboratory values and scoring systems evaluated are summarized in Table 1. Dates of death and liver transplantation were recorded. Baseline laboratory tests were obtained within 3 months of “day 0”. Follow-up laboratory tests were obtained within 3–16 months of “day 0”. The dates of initiation and discontinuation of anticoagulation as well as type of anticoagulation were recorded. The indication and date of TIPS, portosystemic pressure measurements based on patent portal vein below thrombosis (available in 10 patients), stent size and type, utilization of thrombectomy/thrombolysis, and any subsequent TIPS revisions were also recorded.

### 2.3. Imaging Evaluation

Portomesenteric thrombosis was independently evaluated on contrast-enhanced CT or MRI acquired during the portal venous phase by a fellowship-trained abdominal radiologist (V.P). For patients who did not receive therapy, the baseline portomesenteric thrombosis was evaluated on the imaging performed at the time of diagnosis (day 0). Baseline imaging for patients in the TIPS and anticoagulation groups was selected to be the most recent imaging within 3 months prior to day 0. Follow-up imaging was assessed at two points, early follow-up (6–9 months from day 0) and late follow-up (9–16 months from day 0), and were compared with baseline imaging. Studies for early and late follow-up were selected to be at least 3 months apart to reduce bias.

The Yerdel scoring system was used to evaluate the overall portomesenteric thrombosis burden based on the severity of thrombus in the main portal vein as well as SMV extension [8]. The Yerdel grades were grade 1 (less than 50% PV occlusion ± minimal SMV extension), grade 2 (over 50% PV occlusion ± minimal SMV extension), grade 3 (complete PV occlusion + complete proximal SMV occlusion with patent distal SMV segments), and grade 4 (complete occlusion of main PV and SMV, including distal branches).

### 2.4. Outcome Measures

The outcome measure was defined as the overall change in portomesenteric venous system patency on follow-up cross-sectional imaging from baseline using Yerdel grades. The overall change was divided into the following categories: 1, improved patency (i.e., any decrease in Yerdel grade or complete recanalization of PV); 2, stable patency (i.e., no change in Yerdel grade); and 3, worsening patency (i.e., any increase in Yerdel grade). Bleeding complications during the treatment period in the anticoagulation group and 30-day complications after TIPS were reviewed and graded by SIR adverse event classification [18]. Charts were reviewed for the presence of hepatic encephalopathy graded by West Haven criteria until 16 months after diagnosis/treatment. Any new or worsening encephalopathy as well as the grade of encephalopathy were recorded.

### 2.5. Statistical Analysis

Descriptive statistics characterize the baseline data for each cohort of management. Continuous variables were compared using an ANOVA test, ordinal variables compared by the Kruskal–Wallis test, and categorical variables compared with the Fisher exact test. Ordinal logistic regression was performed using changes in the Yerdel grade as dependents. Independent variables included patient management groups, baseline Yerdel grade, and the interval between baseline and follow-up(months). Patient survival was estimated using Kaplan–Meier analysis censored to the date of the last follow-up or curative treatment (liver transplant). Log-rank analysis was performed for comparison of overall survival. Two-tailed *p*-values < 0.05 were considered statistically significant. Statistical analysis was performed using SPSS (IBM SPSS, version 25, SPSS, Chicago, IL, USA).

## 3. Results

### 3.1. Baseline Laboratory and Imaging Characteristics

There were no significant differences in baseline demographics, laboratory values, MELD-Na score, and Child–Pugh class among the three groups (Table 1). The number of patients with a MELD-Na score higher than or equal to 18 was two, one, and two for patients who received no treatment, anticoagulation, and TIPS, respectively (*p* = 0.827). Evaluation of portomesenteric thrombosis burden according to the Yerdel grade at baseline showed no statistically significant differences among the three groups (*p* = 0.721) (Table 1).

### 3.2. Treatment

PVT was the primary indication for all of the patients whose PVT was managed by anticoagulation. The median interval between diagnosis and initiation of anticoagulation (day 0) was 2.2 months (range, 0.2–69.6). The median duration of anticoagulation was 14.6 months (range, 1.3–56.1). Nine (82%) patients were treated with a single anticoagulation agent; of those, three (25%) patients were treated with warfarin, two (17%) were treated with enoxaparin, 2 (17%) were treated with apixaban, and two (17%) were treated with fondaparinux. Two (17%) patients were treated with more than one anticoagulation agent. All patients received therapeutic dose for anticoagulation, and patients who received warfarin achieved therapeutic levels.

The indication for TIPS was PVT for nine patients (64%), variceal bleeding for three patients (21%), and refractory ascites or hydrothorax for one patient (7%). The median interval between TIPS (day 0) and diagnosis was 3.8 months (range, 0.1–39.2). The median pre- and post-TIPS portosystemic gradients were 18.5 mmHg (range, 12–28) and 6.5 mmHg (4.0–11.0), respectively. Covered stents (Gore Viatorr^®^ TIPS Endoprosthesis) were used in 13 patients (93%), and non-covered stents were used in 1patient (7%, treated at an outside hospital with limited records). Two patients received variceal embolization during TIPS performed. One patient with Yerdel grade 4 PVT received a two-staged procedure. First, PV-SMV pharmacomechanical thrombectomy was performed using AngioJet with power pulse spray via percutaneous transhepatic access. TIPS was then created for the recanalized main portal vein. Four patients (29%) required TIPS revision for TIPS stenosis within a median of 39.5 months (range, 29.7–47.4).

### 3.3. Follow-Up Imaging Evaluation

The median interval between early follow-up and baseline was 5.6 months (range, 3.4–8.9). At early follow-up, imaging studies were available for 11 (79%) patients in the TIPS group, for 11 (100%) in the anticoagulation group, and for 21 (78%) in the untreated group. The median interval between late follow-up and baseline imaging was 12.4 months (range, 9.2–16.1). At late-follow-up, imaging studies were available for 9 (64%) patients treated in the TIPS group, 7 (58%) in the anticoagulation group, and 18 (67%) in the untreated group.

**Figure 2:**
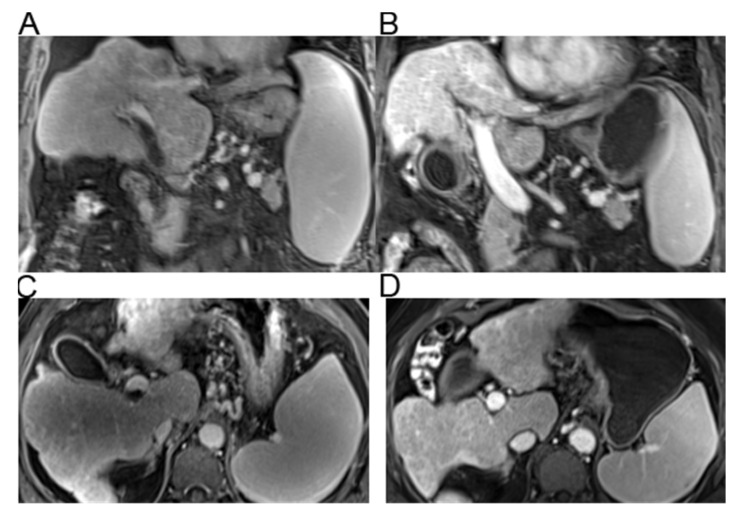
A representative patient with complete resolution of thrombus in the TIPS group. Baseline (**A**) coronal and (**C**) axial imaging 2 months before TIPS showing significant portal vein thrombosis (25–50% occlusion). Follow-up (**B**) coronal and (**D**) axial imaging 6 months after TIPS showing complete resolution of the main portal vein thrombosis.

At early follow-up, overall portosystemic venous system patency based on Yerdel grading improved in eight (72%) TIPS patients, three (27%) anticoagulated patients, and two (10%) untreated patients (*p* = 0.001) (Table 2, Supplemental Figure S1). Only 27% of patients treated with TIPS had no change in portosystemic venous patency compared to a significantly higher percentage of patients in the anticoagulation (73%) and untreated groups (71%) (*p* = 0.042). There was worsening of portosystemic venous patency in 19% of untreated patients compared to none in the TIPS or anticoagulation groups (*p* = 0.164) (Table 2). Complete recanalization of the PV was achieved in 45% of patients in the TIPS group (Figure 2) compared to none in the anticoagulation or untreated groups (*p* < 0.001).

At late follow-up, overall portosystemic venous patency improved in seven (78%) TIPS patients, two (29%) anticoagulated patients, and three (17%) untreated patients (*p* = 0.007) (Table 2, Supplemental Figure S1). Complete recanalization of the PV was achieved in 33% of patients in the TIPS group compared to none in the anticoagulation group and 6% of untreated patients (*p* = 0.007). None of the patients in the TIPS or anticoagulation groups showed worsening patency compared to five (28%) untreated patients, but with no statistically significant difference (*p* = 0.097) (Table 2).

Ordinal logistic regression analysis showed that, at both early and late follow-up, patients in the untreated group had significantly higher associations with increasing Yerdel grades compared to patients in the TIPS group (*p* = 0.001). The odds of Yerdel grade increase for patients in the untreated group was 33.9 and 26.3 times that of patients treated by TIPS at early and late follow-up, respectively (Table 3). Anticoagulation also showed a higher association with increased Yerdel grade compared with TIPS, though only significant at the early follow-up (OR 6.3, *p* = 0.048). The interval between baseline and follow-up studies had no significant association with the change in Yerdel grades at early or late follow-up (*p* = 0.145 and *p* = 0.417, respectively). Univariate analysis showed no significant correlation of Yerdel grade change at early or late follow-up with the baseline thrombosis severity as measured by Yerdel grade (*p* > 0.05).

### 3.4. Follow-Up Laboratory Test and Clinical Outcome

The median interval between the early follow-up laboratory tests and baseline was 6.0 months (range, 3.0–9.0). At early follow-up, laboratory values were available for 10 (71%) patients in the TIPS group, 7 (64%) in the anticoagulation group, and 23 (85%) in the untreated group. The median interval between late follow-up laboratory tests and baseline was 12.1 months (range, 9.1–14.7). At late follow-up, laboratory results were available for 10 (71.4%) patients in the TIPS group, 8 (73%) in the anticoagulation group, and 19 (70.4%) in the untreated group. There were no significant differences in the change in MELD-Na score and Child–Pugh class between the treatment groups (Supplement Table S1). No bleeding complication attributable to the administration of anticoagulant was observed during the treatment period. One patient had an adverse event of moderate severity following TIPS and had intraperitoneal bleeding resulting in hypotension that required blood transfusion. No other TIPS-related adverse events were noted. Five patients (36%) developed new or worsening hepatic encephalopathy within 16 months after TIPS, and severity was limited to mild (grade 1) to moderate (grade 2). The median of the interval between TIPS and onset of encephalopathy was 88 days (6–370). No patients required hospitalization, increased medication, or TIPS closure/restriction because of new or worsening hepatic encephalopathy. In comparison, four patients in the cohort of no treatment (15%) and one patient in the cohort treated by anticoagulation (9%) had new or worsening encephalopathy limited to grade 1–2 within 16 months after diagnosis/treatment. There is no significant difference of encephalopathy percentage among the three cohorts (*p* = 0.262).

Two patients treated with TIPS (14%), one patient treated with anticoagulation (9%), and two patients who received no therapy (7%) received liver transplant. There was no significant difference between liver transplant rates among the three groups (*p* = 0.822). The median overall survival for patients treated with anticoagulation was 66.5 months (Supplemental Figure S2). The median overall survival for patients in TIPS or no treatment groups could not be estimated (median value not reached). There was no statistically significant difference of overall survival among the three groups (*p* = 0.981).

## 4. Discussion

The management of PVT is complicated by increased risk of bleeding and recurrent thrombosis in patients with cirrhosis [1,12]. Anticoagulation is a frequently used therapy to manage PVT. However, a high rate of recurrent thrombosis (36%) has been reported for patients after discontinuing anticoagulation [1]. This is likely related to stagnant flow in the portal vein of a cirrhotic liver. Low blood velocity in the portal vein has been shown as a predictive factor for PVT [4], and anticoagulation does not correct this underlying mechanism for thrombus formation and propagation. Rationally, TIPS, a procedure restoring portal venous outflow, would likely lead to improved treatment of PVT in cirrhosis.

In our study, there was a statistically significant difference in the percentage of patients who showed improved thrombus burden between the study groups. While 72% of patients treated with TIPS showed improved thrombus burden at early follow-up, only 27% of anticoagulated and 10% of untreated patients showed improvement. A similar pattern was noted at late follow-up: 78% of TIPS patients showed improved thrombus burden compared to 29% of anticoagulated and 17% of untreated patients. A comparison of Yerdel grade change showed that anticoagulation had an association with worsening of the portomesenteric thrombosis compared with TIPS, with an odds ratio of 6.3 at early follow-up (*p* = 0.048) and 7.5 at late follow-up (*p* = 0.066). Of note, 88% and 60% of patients managed by TIPS or anticoagulation had available imaging at early and late follow-up, respectively. The imaging response at late follow-up did not reach significance, possibly due to reduced sample size as a result of a lack of follow-up imaging.

A MEDLINE search of the literature available in English from 1 January 1960 to 31 December 2020 using the keywords “portal vein thrombosis”, “anticoagulation”, and “transjugular intrahepatic portosystemic shunt” was performed. No studies were found to directly compare the effectiveness of TIPS to anticoagulation or using no therapy for the management of portomesenteric thrombosis in cirrhotic patients. In a prospective study, Senzolo et al. reported a recanalization rate of 36% for 33 cirrhotic patients with PVT after anticoagulation therapy, 6 of whom also received TIPS [19]. In a randomized trial by Wang et al., there was no significant difference in clinical outcome in patients treated with TIPS alone when comparing TIPS with post-procedural anticoagulation [20]. The study concluded that TIPS placement alone can achieve a high persistent recanalization rate, even without anticoagulation. Findings in our study are consistent with literature reports and support the value of TIPS as mono-therapy in the management of non-tumoral portal vein thrombosis in patients with cirrhosis, particularly for those who did not respond to anticoagulation or for whom anticoagulation is contraindicated.

PVT in untreated cirrhotic patients may progress, but spontaneous recanalization is also possible, with a reported rate of 25.7% [1]. In our study, spontaneous decrease in the thrombus burden was observed in 10% of untreated patients at early follow-up and 17% at late follow-up (Table 2). Of note, the median interval of diagnosis to treatment was 2.0 (0.2–69.6) months for the anticoagulation cohort and 3.8 (0.1–39.2) months for the TIPS cohort. In order to eliminate the effect of spontaneous interval change in thrombus burden in patients managed by anticoagulation or TIPS, day 0 in the two treatment groups was defined as the date of intervention instead of date of diagnosis.

At baseline, patients treated with TIPS and anticoagulation had no significant differences in laboratory tests compared with untreated patients (Table 1). The baseline thrombosis burden was also similar among the treatment groups. In our study, two patients (8%) in the untreated group and one patient (11%) in the anticoagulation group had MELD scores higher than or equal to 18, which suggest that most of these patients with lower MELD scores could potentially be TIPS candidates. As a result, it is reasonable that untreated patients could be candidates for management with anticoagulation or TIPS to restore portomesenteric venous patency, particularly in patients who are transplant candidates.

No bleeding complications attributable to anticoagulation were found in our study cohort. This is consistent with previous literature reports. In a study of 81 patients treated with anticoagulants, Pettinari et al. reported that anticoagulation was an independent factor associated with longer survival at a similar bleeding complication rate compared with untreated patients [1]. Except for one moderate adverse event that required blood transfusion, no other adverse events occurred for patients managed by TIPS in our study. The rate of encephalopathy after TIPS was 36%, in the range of previous reports [21] and with no significant difference compared to the patients treated by anticoagulation alone or no treatment. None of the patients with encephalopathy required hospitalization or escalation of therapy. The overall survival for the three groups was similar. These observations suggested the safety of management with TIPS and anticoagulation.

There are several limitations of this study. First, there was no randomization of the treatment group because of the retrospective nature in this study. The baseline demographics, lab values, and imaging evaluation were not significantly different. However, the relatively small cohort size possibly made the study not powerful enough to show any difference in the baseline PVT among different treatment groups. Univariate analysis showed that the baseline PVT burden had no significant association with thrombosis change at either early or late follow-up. Multivariate analysis cannot be reliably performed due to cohort size; thus, selection bias may still be present. Second, the follow-up interval of each patient also varied. Thus, we divided the follow-up studies into early (3–9 month) and late (9–16 months) to limit the impact of differences in follow-up intervals. Logistic regression analysis also showed no significant contribution from the follow-up interval to the change in portomesenteric burden. Third, the relatively small cohort size made the study not powerful enough to observe any difference in transplant rate and overall survival. Lastly, there were missing studies at follow-up due to the retrospective nature, which also limited longer follow-up. A prospective study with longer follow-up would be needed to compare the clinical outcomes of PVT management.

## 5. Conclusions

In conclusion, both TIPS and anticoagulation are safe and effective therapies to manage non-tumoral portomesenteric thrombosis in patients with cirrhosis. Patients treated with TIPS had a higher association with overall thrombosis burden reduction compared to anticoagulation or no therapy groups. Our results support the value of TIPS in preserving the portal vein in these patients and suggest that TIPS should be considered for cirrhotic patients with non-tumoral portal vein thrombosis, particularly for those whom anticoagulation failed and for potential candidates of liver transplantation. Due to the retrospective nature and relatively small cohort size, the benefit of TIPS in the management of PVT in patients with cirrhosis may need to be further validated by larger-scale prospective studies.

## Figures and Tables

**Table 1:** Baseline characteristics of the study cohort.

	TIPS (14)	Anticoagulation (11)	No Treatment (27)	*p*
**Demographics**				
Age (years) *	58.2 ± 7.0	62.4 ± 10.5	60.7 ± 12.6	0.630
Male sex	12 (86%)	10 (91%)	18 (67%)	0.229
BMI *	30.9 ± 6.4	29.5 ± 4.1	28.9 ± 9.1	0.729
Alcohol use	3 (21%)	3 (27%)	10 (37%)	0.623
Ascites	10 (71%)	6 (55%)	16 (59%)	0.700
Esophageal varices	14 (100%)	11 (100%)	25 (93%)	0.715
Gastric varices	12 (86%)	11 (100%)	23 (85%)	0.527
**Laboratory Tests and Scores**				
Platelet count *	73.6 ± 67.4	117.8 ± 111.7	80.8 ± 66.1	0.390
Total Bilirubin *	2.6 ± 1.9	1.8 ± 1.1	1.6 ± 0.9	0.095
INR *	1.33 ± 0.25	1.28 ± 0.16	1.29 ± 0.19	0.775
MELD-Na *	13.9 ± 3.5	14.6 ± 4.4	12.0 ± 3.6	0.143
C-P class				0.176
A	4 (31%)	6 (67%)	10 (42%)	
B	7 (54%)	3 (33%)	14 (58%)	
C	2 (15%)	0	0	
MELD-NA ≥ 18	2 (15%)	1 (11%)	2 (8%)	0.827
Platelet < 50,000	6 (50%)	2 (22%)	7 (30%)	0.442
**Imaging Assessment**				
Yerdel Grade				0.721
Grade I	7 (50%)	3 (27%)	13 (48%)	
Grade II	5 (36%)	7 (64%)	11 (41%)	
Grade III	1 (7%)	1 (9%)	3 (11%)	
Grade IV	1 (7%)	0	0	

* Data are mean ± standard deviation; all other data are number and percentage (%) of patients. INR: International Normalized Ratio. MELD: Model for End-stage Liver Disease.

**Table 2:** Overall change in portomesenteric venous system patency on follow-up cross-sectional imaging from baseline using Yerdel grades.

	TIPS (14)	Anticoagulation (11)	No Therapy (27)	*p* *
Early follow-up				0.002
Improved	8 (72%)	3 (27%)	2 (10%)	0.001
Stable	3 (27%)	8 (73%)	15 (71%)	0.042
Worse	0 (0%)	0 (0%)	4 (19%)	0.164
Late follow-up				0.015
Improved	7 (78%)	2 (29%)	3 (17%)	0.007
Stable	2 (22%)	5 (71%)	10 (56%)	0.167
Worse	0 (0%)	0 (0%)	5 (28%)	0.097

* Determined with Fisher exact test.

**Table 3:** Univariate ordinal logistic regression analysis of Yerdel grade worsening.

Yerdel Grade Worsening		OR (95% CI)	*p*
**Early Follow-up**	No therapy vs. TIPS	33.9 (4.7–245.7)	0.001
	Anticoagulation vs. TIPS	6.3 (1.0–39.0)	0.048
	Baseline to follow-up interval	0.7 (0.5–1.1)	0.145
**Late Follow-up**	No therapy vs. TIPS	26.3 (3.5–196.2)	0.001
	Anticoagulation vs. TIPS	7.5 (0.9–63.8)	0.066
	Baseline to follow-up interval	0.9 (0.6–1.2)	0.417

## Data Availability

The data presented in this study are available upon request from the corresponding author. The data are not publicly available due to privacy and ethical policy.

## Supplementary Materials

The following are available online at https://www.mdpi.com/article/10.3390/jcm10112316/s1: Figure S1: Overall change in portomesenteric venous patency at (a) early and (b) late follow-up. Figure S2: Kaplan–Meier survival curve for patients with portomensentric thrombosis. The survival for patients managed by TIPS, anticoagulation, or no treatment are represented by green, red, and blue curves, respectively. Table S1: Changes in MELD score and Child–Pughe class at follow-up.
