# Supplementary material for: Comparison of Non-Tumoral Portal Vein Thrombosis Management in Cirrhotic Patients: TIPS Versus Anticoagulation Versus No Treatment
Source: J Clin Med. 2021 May 26;10(11):2316. doi: 10.3390/jcm10112316 (PMC8198761; doi:10.3390/jcm10112316)
Supplement: Supplementary file 1 [file jcm-10-02316-s001.zip › jcm-1202230-supplementary.pdf]

Supplementary Materials:

Supplemental Figure S1. Overall change of portomesenteric venous patency at (A) early and (B) late follow-up.

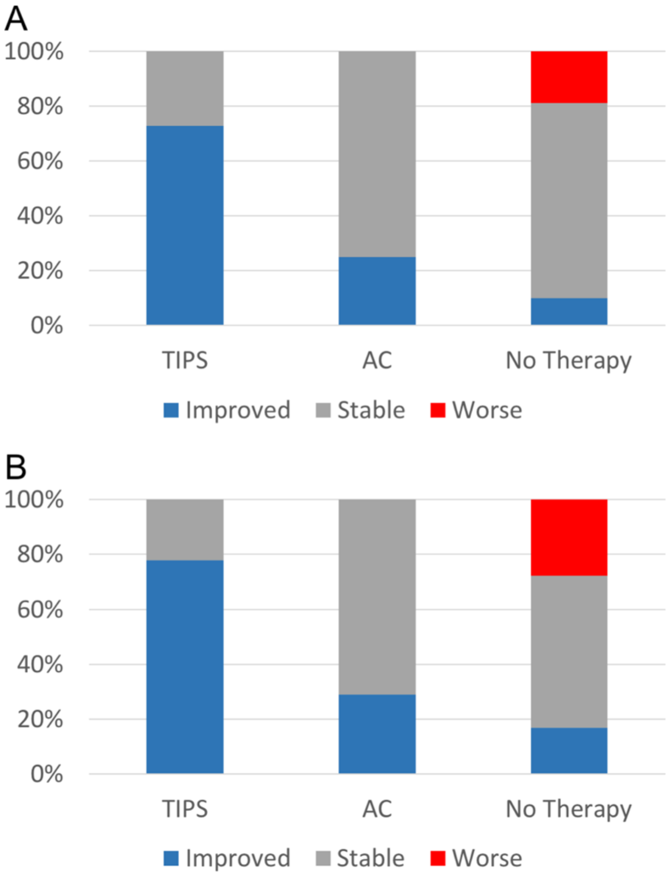

Supplemental Figure S2. Kaplan-Meier survival curve for the patients with portomensentric thrombosis. The survival for patients managed by TIPS, anticoagulation or no treatment are represented by green, red and blue curves, respectively.

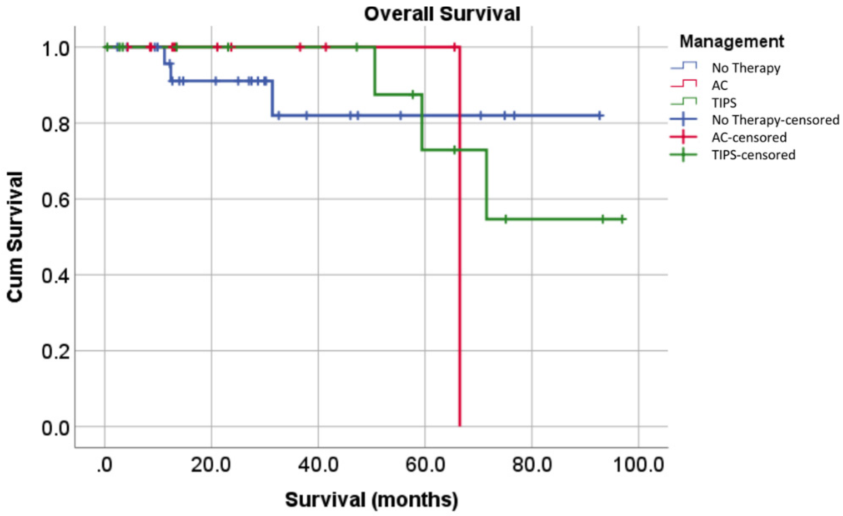

# at risk/Mo	0	20.0	40.0	60.0	80.0	100.0
TIPS	14	10	9	5	2	0
AC	11	6	3	2	0	0
No therapy	27	17	7	4	1	0

Supplement Table S1. Changes of MELD score and Child-Pughe class at follow-up.

**Early follow-up**	**TIPS**	**Anticoagulation**	**No Treatment**	**P***
MELD-Na change	0 (-7 – 12)	-1.5 (-6 – 7)	0.5 (-9 – 4)	0.681
Child-Pughe class change	0 (-2 – 1)	0 (0 – 1)	0 (-2 – 1)	0.457
**Late follow-up**	**TIPS**	**Anticoagulation**	**No Treatment**	**p**
MELD-Na change	0 (-4 – 6)	-1 (-5 – 5)	1 (-6 – 32)	0.672
Child-Pughe class change	0 (-2 – 1)	0 (0 – 0)	0 (-1 – 1)	0.744
Values are median and (range) *Kruskal Wallis Test				
